# Appropriateness of chronic asthma management and medication adherence in patients visiting ambulatory clinic of Gondar University Hospital: a cross-sectional study

**DOI:** 10.1186/s40413-018-0196-1

**Published:** 2018-08-10

**Authors:** Zelalem Tilahun Tesfaye, Nebeyu Tsegu Gebreselase, Boressa Adugna Horsa

**Affiliations:** 1 Department of pharmacology and clinical pharmacy, school of pharmacy, college of health sciences, Addis Ababa University, Addis Ababa, Ethiopia; 2 Oromia Regional State, East Hararghe Zone, Deder General Hospital, Deder, Ethiopia; 3 Department of clinical pharmacy, school of pharmacy, College of Medicine and Health Sciences, University of Gondar, Gondar, Ethiopia

**Keywords:** Inhalation corticosteroids, Stepwise asthma therapy, Beclomethasone, Gondar Univesity hospital

## Abstract

**Background:**

Although asthma cannot be cured, appropriate management can ensure adequate control of the disease, prevent disease progression and even reverse the illness, enabling people to enjoy good quality of life. Predisposing factors for inappropriate asthma management, including limited diagnostic options and inadequate supply of medications, are features of health institutions in developing countries like Ethiopia. This study was launched to determine the appropriateness of asthma management in patients visiting ambulatory clinic of the Gondar University Hospital.

**Methods:**

Cross-sectional study was conducted on asthmatic patients who were on chronic follow-up at Gondar University Hospital. Data were collected by review of patients’ medical records and through a semi-structured questionnaire. The Global Initiative for Asthma guideline was used as a reference for determining the  appropriateness of asthma management whereas the eight-item Morisky medication adherence scale (MMAS-8) was used to collect data on patients’ adherence to asthma medications.

**Results:**

The study participants’ ages ranged from 20 to 80 years with a mean age of 49.3 ± 13.6 years. Mild asthma showed a slight predominance in frequency accounting for 38.7% of cases. Asthma management was found to be inappropriate in 52.0% of the patients. Inappropriateness of therapy is attributed to incorrect dosing of medications, addition of unnecessary medications and omission of necessary medications. Patients who had moderate asthma were more likely to receive appropriate treatment [AOR = 728: 63.2, 8386.06], whereas having a treatment regimen of beclomethasone with salbutamol was found to be predictor of inappropriate treatment [AOR = 0.004: 0.001, 0.07]. More than half (56.7%) of the study subjects reported to have high adherence to their medications. Having no formal education was a predictor of low adherence to asthma medications [AOR = 0.051: 0.003, 0.978] whereas, increased monthly income was found to have a positive association with adherence [AOR = 1.923: 1.037, 3.566].

**Discussion:**

High prevalence of inappropriate therapy in this study may be attributed primarily to limited accessibility of asthma medications, as 86% of the patients received medium dose beclomethasone with salbutamol for exacerbations despite being at different severity of asthma and level of control.

**Conclusion:**

The findings of the study showed more than half of asthmatic patients received inappropriate treatment. Nevertheless, a larger proportion of the patients claimed to be highly adherent to their medications.

## Background

According to the “National Institutes of Health (NIH) Expert Panel Report 3 (EPR-3): guidelines for the diagnosis and management of asthma”, asthma is defined as a chronic inflammatory disorder of the airways in which many cells and cellular elements play a role, in particular, mast cells, eosinophils, T lymphocytes, neutrophils, and epithelial cells [1].

Asthma is now one of the most common chronic diseases affecting an estimated 300 million people worldwide. The prevalence of asthma increased significantly over the second half of the last century, especially in modernized societies [2]. Although there are no data concerning the prevalence of asthma among adults in Ethiopia, the 2014 Global Asthma Report estimated asthma symptoms to be 9.1% in the adolescent population [3], while the International Study of Asthma and Allergies in Childhood (ISAAC) study estimated asthma symptom prevalence of 9.9% in the pediatric population of Ethiopia [4]. The burden of asthma is significant and its impact includes reduced quality of life, lost productivity, increased health care costs, the risk of hospitalization and even death [5].

Although asthma cannot be cured, appropriate management can ensure adequate control of the disease, prevent disease progression and even reverse the illness, enabling people to enjoy good quality of life [6]. The guidelines recommend a stepwise approach for treatment of asthma [1, 6]. Treatment is started at the step most appropriate to the initial severity of the asthma, with a goal of achieving early control of symptoms and optimizing respiratory function. Control is achieved by stepping up treatment as necessary and stepping down when control is good [1, 6, 7].

There are many factors that determine appropriateness of asthma management: including access to adequate treatment, medication availability, cost of the medication, training of primary healthcare professionals and availability of adequate diagnostic services [8–10]. In the less developed countries, under-recognition and under-treatment limit the success of asthma management. Moreover, a low rate of distribution and implementation of national and international guidelines may hinder achievement of the global initiative for asthma control goals [8].

The cost and availability of medications for asthma vary widely and this represents an important barrier for effective management in some low and middle income countries, as access to adequate treatment is essential for better management of asthma [10, 11].

Predisposing factors for inappropriate asthma management including limited diagnostic options and unavailability of medications are features of health institutions in developing countries like Ethiopia. Furthermore, low socioeconomic status of the community around Gondar may increase the risk of inappropriate management and low medication adherence in asthma patients treated in Gondar University Hospital (GUH). Despite these risk factors, no study has been previously conducted in this area to quantify the extent of the problem. This study was launched to determine the appropriateness of asthma management in patients visiting ambulatory clinic of the GUH.

## Methods

A cross-sectional study was conducted at the ambulatory clinic of GUH. The hospital, which was established in 1954, now has 400 beds and gives health services to the population in the northwestern part of the country [12]. The study was conducted from April 1 to May 31, 2017.

The study included asthmatic patients who were on chronic follow-up at GUH. Sample size was estimated using single population proportion formula. After adjusting for sample size based on the total population of patients scheduled to visit over the study period, and adding 10% contingency, the final sample size was calculated to be 162. Systematic random sampling was used to draw study subjects from the population.

Data were collected by review of patient medical records and through a semi-structured questionnaire. Data collected through medical record review included patient specific data such as age, sex, duration since asthma diagnosis, severity of asthma, and types of asthma medications. The semi-structured questionnaire was used to collect socio-demographic characteristics such as patients’ income, marital status, level of education etc.; and to collect data on patients’ adherence to asthma medications using the eight-item Morisky medication adherence scale (MMAS-8). This scale produces scores ranging from 0 to 8 where the score of 0 indicates high adherence, scores 1 and 2 indicate intermediate adherence, and scores 3 to 8 indicate low adherence. The questionnaire was autonomously filled in by the patients unless they were unable to read in which case the data collector would read the questions for the subjects and record their responses.

The Global Initiative for Asthma (GINA) guideline was used as a reference for determining the appropriateness of asthma management. Thus, the management of asthma was reported to be appropriate if the following conditions were fulfilled; 1) patients had been treated according to GINA recommendations of stepwise approach to asthma treatment, 2) adjustment had been made for patients visiting for follow-up according to GINA recommendations after review of response, 3) consideration to individual patient profiles (such as age, comorbidities, etc.) had been made in selection of therapeutic regimens. Asthma management was reported as inappropriate if it did not meet at least one of the three conditions.

The data were entered into IBM SPSS Statistics 24® software. Binary logistic analysis was used to identify association between the independent and outcome variables where *p* < 0.05 was considered as statistically significant.

## Results

### Socio-demographic characteristics

Out of 162 patients selected by systematic random sampling, 12 either had incomplete medical records or were not available for filling in the questionnaire on adherence. Thus, data from 150 patients was analyzed, giving us a response rate of 92.6%. The study participants age ranged from 20 to 80 years with mean age of 49.3 ± 13.6 years. Slightly more than half of the respondents (51.3%) were women, and 76.7% of the participants were married. With regard to educational and employment status, 43.3% of the participants did not have formal education while 34.0% were unemployed (Table 1).

**Table 1:** Socio-demographic characteristics of asthmatic patients treated at ambulatory clinic of Gondar University Hospital

Variables	Frequency (%)
Age in years (mean ± SD)	49.3 ± 13.61
< 35	20 (13.3)
35–44	32 (21.3)
45–54	45 (30)
55–64	29 (19.3)
65+	24 (16)
Gender	
Female	77(51.3)
Male	73(48.7)
Level of education	
No formal education	65(43.3)
Primary	27(18)
Secondary	28(18.7)
Higher education and above	30(20)
Marital status	
Single	27(18)
Married	115(76.7)
Divorced	1(0.7)
Widowed	7(4.7)
Residence	
Urban	120(80)
Rural	30(20)
Religion	
Orthodox	128(85.3)
Muslim	15(10)
Protestant	7(4.7)
Employment status	
Farmer	26(17.3)
Business man	15(10)
Government employer	48(32)
Retired	9(6)
Unemployed	51(34)
Other	1(0.7)
Monthly income in ETB* (mean ± SD) (*n* = 138)	1815.94 ± 1278.143
≤ 550	22(15.9)
551–1000	35(25.4)
1001–1550	21(15.2)
1551–3000	38(27.5)
3001+	22(15.9)
Distance from the hospital in kilometers (mean ± SD)	12.4 ± 21.98
≤ 5	106(70.7)
6–15	6(4)
16–25	16(10.7)
26–35	6(4)
36+	16(10.7)

Note: *ETB* Ethiopian Birr, *SD* Standard deviation

*ETB/USD exchange rate at the time of the study; 1 ETB = 0.056USD

### Disease characteristics

**Fig. 1:**
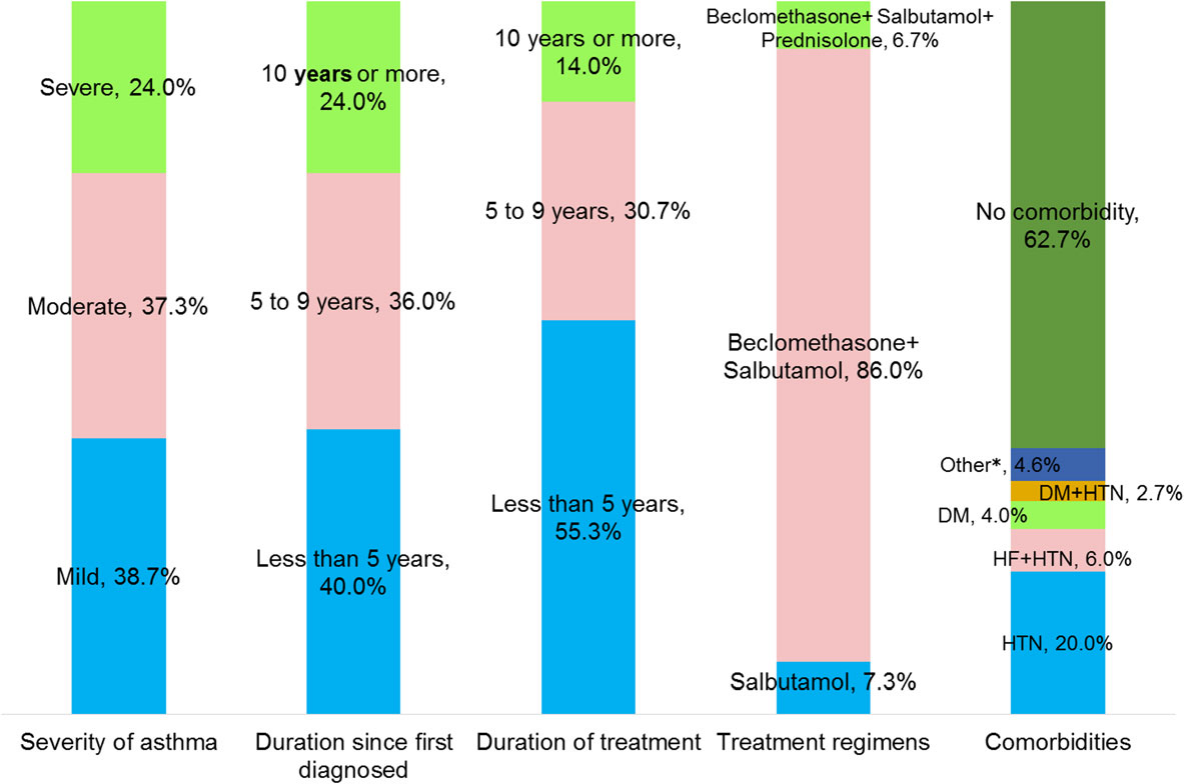
Disease characteristics of chronic asthma patients treated at ambulatory clinic of Gondar University Hospital. Other*, 7 (4.6%) – Rheumatic heart disease, 3; Epilepsy, 1; Glaucoma, 1; Human Immunodeficicecy virus, 1. Abbreviations – DM, Diabetes mellitus; HF, Heart failure; HTN, Hypertension

Mild asthma showed slight predominance in frequency accounting for 38.7% of cases; and 37.3% of the participants had overlaying co-morbidities, hypertension being the most common co-morbidity. As many as 60% of the respondents reported to have been diagnosed as asthmatic before 5 years while about 45% had been on asthma medications for more than 5 years. The most common treatment regimen was twice daily beclomethasone and as needed salbutamol (also known as albuterol), with a prevalence rate of 86% (Fig. 1).

### Appropriateness of asthma management and associated factors

**Fig. 2:**
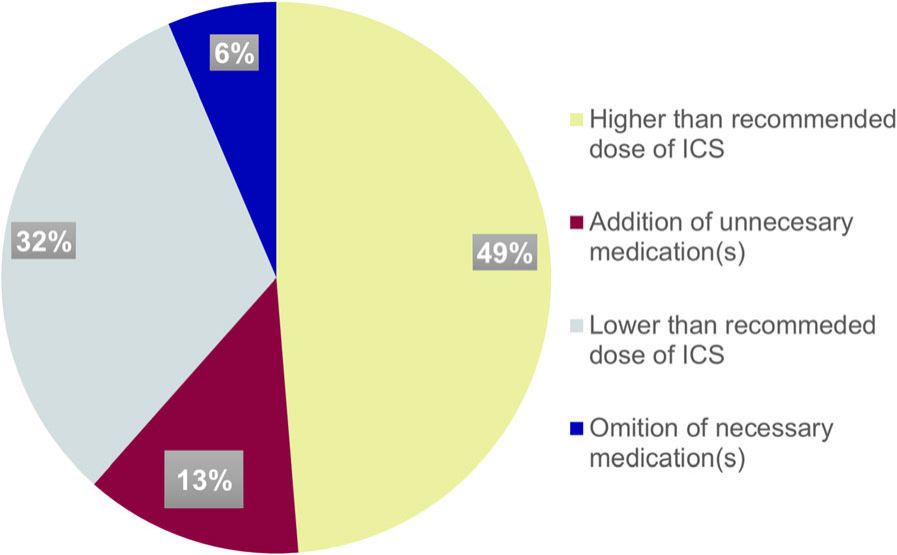
Reasons behind inappropriateness of asthma management in patients treated at ambulatory clinic of Gondar University Hospital

Appropriateness of asthma management was determined based on the GINA guideline for the management of asthma. Accordingly, asthma management was found to be inappropriate in 78 (52.0%) patients. Inappropriateness of therapy is attributed to deviations from GINA recommendations of stepwise approach to asthma management that included: incorrect dosing of inhalation corticosteroids (ICS) depending on asthma symptoms, severity and level of control; failure to consider additional medications such as long acting beta-agonists (LABA) and leukotriene modifiers in some cases of uncontrolled asthma; and addition of medications that should not necessarily be prescribed to control the patient’s symptoms (for example, addition of controller medications for patients who can effectively be treated with relief medications alone, or unjustified addition of oral corticosteroid to patient’s regimen) (Fig. 2).

Univariate and multivariate analyses were performed to identify factors associated with appropriateness of drug therapy. As a result, patients who had moderate asthma were more likely to receive the right treatment [AOR = 728: 63.2–8386.06], whereas having a treatment regimen of beclomethasone with salbutamol was found to be a predictor of inappropriate treatment [AOR = 0.0040: 001, 0.07)] (Table 2).

**Table 2:** Factors associated with appropriateness of asthma management

Determinant factors	Appropriateness of asthma management		COR (95% CI)	AOR (95% CI)
	Appropriate *n* (%)	Inappropriate *n* (%)		
Severity of asthma				
Mild	10 (6.67)	47 (31.3)	0.617 (0.22, 1.663)	3.574(0.41, 31.247)
Moderate	52 (34.67)	2 (1.33)	75.4 (15.46, 367.781)*	728(63.2, 8386.06)*
Severe	10 (6.67)	29 (19.33)	1.00	1.00
Asthma medication				
Salbutamol only	4 (2.67)	0	179,497,207.2 (0.00, −)	50,216,480.6(0.00, −)
Salbutamol + Beclomethasone	59 (39.33)	77 (51.33)	0.085 (0.01, 0.691)*	0.004(0.001, 0.07)*
Salbutamol + Beclomethasone + Prednisolone	9 (6)	1 (0.67)	1.00	1.00

Note: *AOR* Adjusted odds ration, *COR* Crude odds ratio, *CI* Confidence interval

*Bold indicates significant values (*p* < 0.05)

### Patient medication adherence

MMAS-8 was used to determine patients’ adherence to their asthma medications. As a result, more than half (56.7%) of the study subjects reported to be fully adherent to their medications (scored 0 on MMAS-8), whereas 20.7% had intermediate adherence (scored 1–2 on MMAS-8) and the remaining patients (22.7%) had low adherence to their medications (scored 3–8 on MMAS-8).

Both univariate and multivariate analyses showed the presence of association between several predictor variables and patient adherence. For instance, level of education was associated with level of adherence, with having no formal education being negatively associated with adherence to asthma medications [AOR = 0.051: 0.003–0.978]. Furthermore, an increase in patients’ monthly income was found to have positive association with adherence [AOR = 1.923: 1.037, 3.566] (Table 3).

**Table 3:** Factors associated with patients’ adherence to asthma medications

Factors	Patient adherence		COR (95% CI)	AOR (95% CI)
	Adherent: *n* (%)	Non-adherent: *n* (%)		
Age			0.923(0.894, 0.952)*	0.614(0.31, 1.215)
Sex				
Female	40(26.67)	37(24.67)	0.673(0.351, 1.288)	1.415(0.444, 4.504)
Male	45(30)	28(18.67)	1.00	1.00
Level of education				
No formal education	23(15.33)	42(28)	0.061(0.017, 0.223)*	0.051(0.003, 0.978)*
Primary school	14(9.33)	13(8.67)	0.12(0.029, 0.491)*	0.149(0.018, 1.222)
Secondary school	21(14)	7(4.67)	0.333(0.077, 1.447)	0.273(0.038, 1.95)
Higher education	27(18)	3(2)	1.00	1.00
Marital status				
Single	22(14.67)	5(3.33)	26.4(2.571, 271.088)*	0.794(0.029, 21.726)
Married	62(41.33)	53(35.33)	7.019(0.819, 60.167)	1.033(0.065, 16.397)
Divorced	0	1(0.67)	0	0.00(0.00)
Widowed	1(0.67)	6(4)	1.00	1.00
Residence				
Urban	75(50)	45(30)	3.333(1.433, 7.754)*	548,805,661.4(0.00)
Rural	10(6.67)	20(13.33)	1.00	1.00
Religion				
Orthodox	72(48)	56(37.33)	0.214(0.025, 1.832)	0.423(0.033, 5.47)
Muslim	7(4.67)	8(5.33)	0.146(0.014, 1.525)	0.17(0.007, 3.211)
Protestant	6(4)	1(0.67)	1.00	1.00
Monthly income			2.15(1.57, 2.93)*	1.923(1.037, 3.566)*
Severity of asthma				
Mild	3724.67)	20(13.3)	2.16(0.94, 4.96)	1.217(0.343, 4.319)
Moderate	30(20)	24(16)	1.46(0.64, 3.335)*	1.554(0.476, 5.071)
Sever	18(12)	21(14)	1.00	1.00
Co-morbidity				
Present	27(18)	29(19.3)	0.58(0.296, 1.13)	1.598(0.58, 4.398)
Not present	58(38.67)	36(24)	1.00	1.00
Duration since diagnosis			0.942(0.896, 0.991)*	0.884(0.737, 1.061)
Duration of treatment			0.94(0.87,1.007)	1.067(0.86, 1.323)

Note: *AOR* Adjusted odds ration, *COR* Crude odds ratio, *CI* Confidence interval, *ICS* Inhalation corticosteroids

*Bold indicates significant values (*p* < 0.05)

## Discussion

Although asthma is an incurable disease, it can be effectively controlled by medications. Appropriate use of asthma medications is the cornerstone for successful control of the disease progression. This study gives valuable information about appropriateness of asthma management and the extent of patient adherence to asthma medications.

In this study, the management of asthma was found to be inappropriate in 52% of the patients. This figure lies between findings from Italy [10] and Sudan [13] where the prevalence of inappropriate asthma management was 48 and 57.1% respectively. A much higher proportion of inappropriate asthma therapy was reported by a domestic study conducted in Jimma Specialized Hospital in which only 21% of the patients received appropriate treatment according to GINA recommendations [14]. This difference indicates that there is better practice regarding chronic asthma management in GUH as compared to Jimma Specialized Hospital. However, there is still a lot to be improved in our study setting to ensure appropriate asthma management based on the GINA guideline.

In the present study severity of asthma was found to be associated with appropriateness of asthma management where moderate asthma diagnosis was a predictor of appropriate treatment. This association resulted from the fact that most of the patients (86%) received medium dose Beclomethasone with Salbutamol for exacerbations, which coincided with the recommended treatment for patients presented with moderate asthma. In contrast, Beclomethasone with as needed Salbutamol, which was the prescribed regimen for 86% of the patients, was a predictor of inappropriate therapy. This regimen has been over-prescribed whether it is in line with GINA recommendations or not. It appears that beclomethasone was the only corticosteroid prescribed for all patients on ICS. This is because it is the only ICS available in the hospital pharmacy and the assumption by physicians that patients could not afford to buy other ICS or ICS/LABA combinations from community pharmacies.

In this study, 43.3% of the participants were non-adherent to their asthma medications. This finding is slightly higher than that of an Australian study which reported about 38% of the patients to be non-adherent [15]. This difference may be due to differences in socio-economic status and awareness of the patients in the two study areas. Non-adherence to the regular asthma therapy is associated with uncontrolled asthma and increasing rate of hospitalization [16]. Thus, healthcare providers should provide adequate counseling for chronic asthma patients to ensure effective asthma control.

Having no formal education was associated with non-adherence to asthma medications in our study, which agrees with the report of a study by Rifaat et al. [17]. This finding can be explained by lower level of awareness and perception on the importance of asthma medications in less educated patients. Similarly, low monthly income was a predictor of non-adherence. Patients with low income may not be able to purchase adequate medications or they may not afford transportation to the hospital to get a regular medication refill.

The limitations of the study should not be overlooked. Since this is a cross-sectional study, it is not possible to establish temporal relationship between cause and effect. Thus, the effect of inappropriate treatment and low adherence on asthma control was not explored. It is also important to note that the study included a fairly small number of study subjects and did not cover pharmaco-economic aspects of therapy such as medication cost and availability. Despite these limitations, the findings of this study can be useful input for understanding the extent of the problem and taking measures to improve the practice of asthma management.

## Conclusion

The findings of the study showed more than half of asthmatic patients received inappropriate treatment. Nevertheless, a larger proportion of the patients claimed to be highly adherent to their medications. Moderate asthma severity was associated with appropriate therapy, whereas low educational background and low income were found to be predictors of non-adherence to asthma medications.

## Abbreviations

AOR: Adjusted odds ratio
CI: Confidence interval
COR: Crude odds ratio
EPR-3: Expert Panel Report 3
ETB: Ethiopian Birr
GINA: Global initiative for asthma
GUH: Gondar University Hospital
ICS: Inhalation corticosteroids
NIH: National Institutes of Health
SD: Standard deviation
